# Research on the Changes in Distribution and Habitat Suitability of the Chinese Red Panda Population

**DOI:** 10.3390/ani14030424

**Published:** 2024-01-28

**Authors:** Tao Ruan, Wei Wei, Zejun Zhang, Hong Zhou

**Affiliations:** 1 College of Giant Panda, China West Normal University, Nanchong 637009, China; ruantao0716@163.com (T.R.); weidamon@163.com (W.W.); zhangzejun66@163.com (Z.Z.); 2 Liziping Giant Panda’s Ecology and Conservation Observation and Research Station of Sichuan Province, Nanchong 637009, China

**Keywords:** Chinese red pandas, species distribution model, habitat suitability, distribution shrinkage, conservation strategy

## Abstract

**Simple Summary:**

The study emphasizes the importance of species–habitat dynamic research in shaping effective habitat protection and management strategies. Current, research on Chinese red panda habitats is limited to single-period analysis, thereby hindering the formulation of comprehensive conservation strategies. The study employs habitat suitability simulations across different time scales, quantifying trends in habitat quality changes, and analyzing the reasons for suitability changes. The results show an overall increase in habitat suitability for the Chinese red panda, but a decline in habitat suitability in the central part of Liangshan is observed. The decline in habitat suitability is attributed to climate change and human interference. Additionally, the local extinction of the isolated populations in the Minshan Mountains is identified as the primary cause of the distribution retreat rather than a decrease in habitat quality being identified as such. These findings provide a scientific basis for developing conservation and management strategies for Chinese red pandas at different scales.

**Abstract:**

The study of the dynamics of species habitat is of great significance for maintaining or adjusting the current habitat protection management strategy. However, the current research on the Chinese red panda’s habitat is limited to the analysis of a single period, which makes it difficult to quantify the changes in its habitat on a temporal scale and greatly hinders the formulation of the overall protection and management strategies that are to be used for the Chinese red panda. This study simulated habitat suitability at different temporal scales to quantify the trend of changes in habitat quality and analyzed the reasons for the changes in habitat suitability in certain regions. The results showed that the overall suitability of the Chinese red panda’s habitat increased and that the area of suitable habitats expanded. Suitable Chinese red panda habitats in the mountains of Qionglai (1662.73 km^2^), Daxiangling (230.30 km^2^), Xiaoxiangling (549.47 km^2^), and Liangshan (50.39 km^2^) increased by a total of 2452.89 km^2^. The suitability of habitats in the central part of the Liangshan Mountains has declined significantly, which is positively correlated with changes in temperature seasonality (BIO4, R = 0.18) and negatively correlated with changes in annual average temperature (BIO1, R = −0.03) as well as changes in the proportion of farmland (FARMLAND, R = −0.14). The local extinction of isolated populations of Chinese red pandas in the Minshan Mountains is the main factor leading to their distribution retreat rather than a decrease in habitat quality. The research results help us to provide a scientific basis for the formulation of conservation and management strategies for Chinese red pandas at different scales.

## 1. Instruction

There are two extant species of red panda, namely that of the Himalayan red panda (*A. fulgens*) and that of the Chinese red panda (*Ailurus styani*) [1], which are distributed in the Himalayas and adjacent areas including Nepal, India, Bhutan, Sikkim, Myanmar, and Southwestern China’s Sichuan, Yunnan, and Tibetan provinces. The westernmost point of the distribution of the Chinese red panda is in the Mugu region of Western Nepal (82° E), the easternmost point is in the upper Minjiang River in Sichuan (104° E), the southernmost point is in the southern part of the Gaoligong Mountains on the Sino–Myanmar border (25° N), and the northernmost point is in the Minshan mountains in the upper Minjiang River (33° N) [2]. In China, the Himalayan red panda is distributed in the region to the west of the Yarlung Zangbo River in Tibet, while the Chinese red panda is distributed in Sichuan, Yunnan, and the region to the east of the Yarlung Zangbo River in Tibet, with most of them distributed in Sichuan Province. However, the results of the “fourth national giant panda survey” showed that, except for a few Chinese red panda individuals remaining in the Mianzhu and Shehong areas at the southernmost end of the Minshan mountains, Chinese red pandas may have become extinct in other areas of the Minshan mountains (such as Pingwu, Qingchuan, etc.) [3,4]. Wei et al. (1999) conducted a survey on the population status of wild Chinese red pandas in Sichuan and Yunnan, China, and found that there were approximately 3000–4000 individuals in Sichuan and 1600–2000 individuals in Yunnan. However, existing data show that over the past few decades the distribution range of Chinese red pandas in China may have shrunk significantly and the population size has also declined sharply [5].

The Chinese red panda is a typical herbivorous carnivore, but it has poor energy utilization from bamboo, resulting in a low digestion rate. To meet its daily energy requirements, the Chinese red panda needs to consume a large amount of bamboo and related foods to maximize its energy intake rate [6]. Chinese red pandas exhibit seasonal activity patterns, with higher activity rates in spring, summer, and autumn, and lower rates in winter. Additionally, they have crepuscular activity habits [7]. The Chinese red panda is a mountain forest-dwelling animal; research has found that Chinese red pandas in different regions have varying habitat preferences, which are primarily influenced by human disturbances, climate change, and specific preferences for survival resources [8,9,10]. Forest logging not only causes habitat fragmentation and the reduction of the available habitat area but also directly affects the migration of individuals between different habitat patches and may lead to population extinction [11,12]. According to Wei et al. (1999), 22 large-scale forest enterprises in Sichuan caused the loss of 3597.9 km^2^ of the Chinese red panda’s habitat area over 25 years [5]. In Langtang National Park, due to overgrazing, more than 60% of the Chinese red panda’s habitat was severely damaged, and livestock grazing activities were the main cause of death of Chinese red panda cubs in the area [13]. As a good fur animal that combines both economic value and aesthetic appeal, hunting and trade are also important reasons for the decline in the species population [5]. It has been reported that Sichuan Mianning purchased 19 Chinese red panda furs in 1979–1981, and Tibet purchased more than 200 Chinese red panda furs in the 1970s. An incident of illegal hunting and selling wild Chinese red pandas occurred in Leshan City, Sichuan Province, in 2022, which involved 63 Chinese red pandas. (https://m.gmw.cn/2022-11/03/content_1303185356.htm, last retrieved on 15 December 2023). In some places in Nepal, locals used to catch wild Chinese red pandas to sell to Western zoos [2,14].

Due to human activities such as poaching and habitat destruction, wild Chinese red pandas have gradually become endangered, sparking widespread concern in the international community. The International Union for the Conservation of Nature (IUCN) has listed Chinese red panda as “Endangered”, and the Convention on International Trade in Endangered Species of Wild Fauna and Flora (CITES) has listed it in Appendix I [15]. China passed the Wildlife Protection Law in 1988, and Chinese red pandas and other species were listed as a national second-level key protected wild animal. According to incomplete statistics, by 2020, China had established 50 nature reserves in the Chinese red panda’s distribution area within a year, including 37 in Sichuan, 7 in Yunnan, and 6 in Tibet [16]. However, research on wild Chinese red pandas is currently limited to aspects such as activity rhythms, habitat selection, foraging strategies, and suitable habitat distribution [8,9,17,18,19]. Compared with the celebrity species that is the giant panda, there is less research on wild Chinese red pandas, and the ecological and biological knowledge of this species is insufficient in terms of depth and breadth. Conducting research on this species is of great significance in addressing the limitations of existing studies.

Habitat quality evaluation is not only an important means of assessing the survival status of Chinese red pandas but also an important basis for formulating habitat protection and management strategies; therefore, it is highly valued. Species distribution models (SDMs), such as maximum entropy model (MaxEnt) and random forest model (RF), are represent an important tool for evaluating the habitat quality of wild animals and have high accuracy and stability. It has been widely used by researchers for habitat quality assessment-related work [20,21]. The current quality assessment of the Chinese red panda’s habitat mostly obtains habitat status information from the landscape scale, aiming to understand the distribution and fragmentation degree of the Chinese red panda’s habitat, and then propose protection and management suggestions for habitat distribution [22,23,24]. Understanding the change in the trend of Chinese red panda habitat quality is the premise for formulating macro-management strategies for habitat. However, there is no research on the spatiotemporal changes of the Chinese red panda’s habitat at present. In the current context, characterized by adverse factors such as climate change and human activities coexisting with conservation measures, understanding how the suitability of Chinese red panda habitats is changing and identifying the factors influencing these changes has become a key issue in the conservation and management of Chinese red panda populations and their habitats. This study is based on the distribution of Chinese red panda populations at different time scales and uses species distribution models to compare and analyze the habitat quality of Chinese red pandas at different time scales, and clarify its changes and analyze the causes. It provides a reference for guiding the conservation work of species, and also provides a new idea for evaluating the effectiveness of biodiversity conservation in the future.

## 2. Material and Methods

### 2.1. Study Area and Species Distribution Data

This study takes the mountains where the Chinese red panda is concentrated as the research area. This area is not only a global biodiversity hotspot but also the only area where the Chinese red panda and giant panda coexist [25]. Due to the barriers of mountains and rivers, this area is divided into five independent mountains, namely the Minshan mountains, Qionglai mountains, Xiaoxiangling mountains, Daxiangling mountains and Liangshan mountains. There are two main reasons for choosing the five mountains with sympatric distribution as the research area. On the one hand, it can use the data of sympatric Chinese red pandas in the database of multiple giant panda resource surveys to analyze the quality and change in the Chinese red panda’s habitat. On the other hand, it can also reflect whether the sympatric species population and habitat have been effectively restored due to the umbrella effect under the implementation of China’s long-term giant panda population and habitat protection policies.

**Figure 1:**
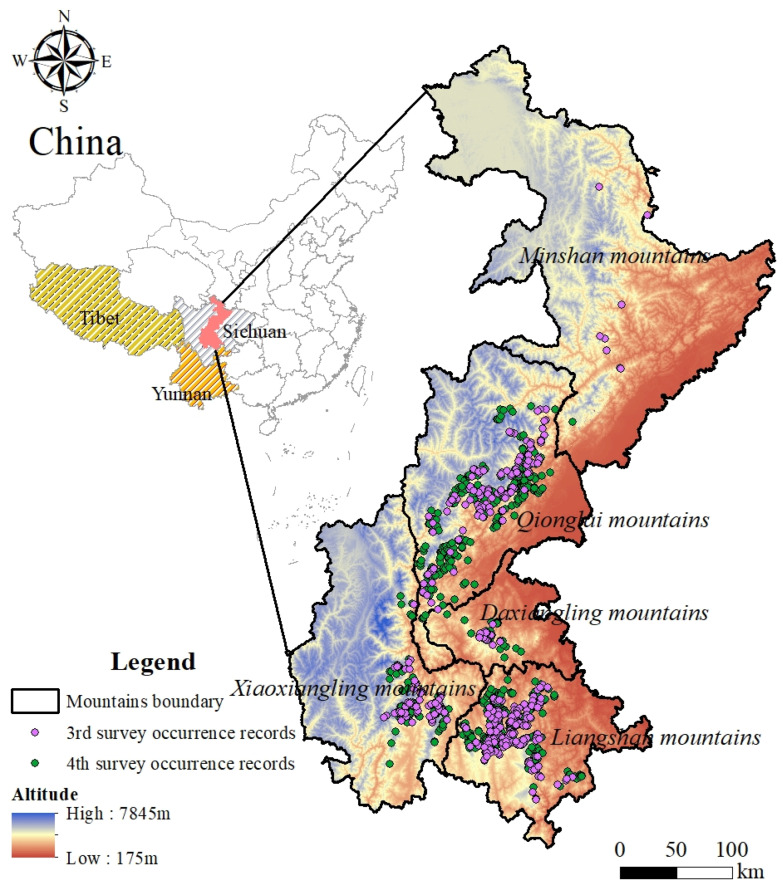
Overview of the study area and distribution of Chinese red panda traces.

To monitor the survival status of giant panda populations, China has completed four surveys of giant panda population resources in the research area. Among them, the third and fourth surveys (3rd survey: 1998–2002; 4th survey: 2011–2014) [3,26] recorded in detail the traces (such as feces, hair and foraging signs) of sympatric species at the sites where giant pandas were distributed, which provided the necessary data support for this study to carry out dynamic assessment of Chinese red panda habitat quality. A total of 378 records of Chinese red panda presence were obtained during the third survey period, and the distribution of trace sites were extended from the northern part of the Minshan mountains to the southern part of the Liangshan mountains. A total of 726 records of the Chinese red panda’s presence were obtained during the fourth survey period. Within the Minshan mountains, except for the border area with the Qionglai mountains, no traces of Chinese red pandas were found (Figure 1).

### 2.2. Environmental Factors

This study selected three major categories of ecological factors affecting the distribution of Chinese red panda populations, namely climatic factors, land use factors, and topographic factors, as the environmental variables for building the model (Table 1).

Climatic factors: Climate change has been proven to be the main driving factor for the current species distribution pattern and is an important environmental factor for assessing habitat quality [27,28]. Bioclimatic variables were selected to participate in the assessment of Chinese red panda habitat suitability. Monthly temperature and precipitation measurement datasets came from the National Tibetan Plateau Scientific Data Center. The data from 2000 and 2010 were selected to describe the climatic conditions at different time periods because these two time points were consistent with the giant panda survey time and could reflect the climatic conditions at the time of the survey. Both datasets provided monthly grid data with a spatial resolution of 0.0083° (about 1 km). Based on these data, 19 bioclimatic variables were created using the bioclim function in the R package dismo (https://CRAN.R-project.org/package=dismo, last retrieved on 13 December 2023) following the steps described by Hijmans et al. [29].

Land use factors: Animals have different response mechanisms to different land use types, either avoiding residential areas or relying on water bodies to build nests [30,31]. Therefore, we included the land use types of the study area into the environmental factor dataset. The data came from the Chinese Academy of Sciences Resource and Environmental Science and Data Center (CNLUCC; http://www.resdc.cn, last retrieved on 26 December 2023) providing grid data with a spatial resolution of 1 km. The area coverage ratio of six different land use types (farmland, forest, grassland, water, residential land, and other land) were included in the dataset. The land use layers of 2000 and 2010 were overlaid to the study area, and the proportion of each land use type was calculated. Road data came from the first edition of the global road open access dataset (gROADS).

Topographic factors: Topographic factors such as elevation, slope, and distance to roads can accurately describe the environment where species live and are commonly used data in the habitat suitability evaluation of large- and medium-sized wild animals. Elevation data came from the 30 m digital elevation model provided by NASA’s shuttle radar topography mission (SRTM), and slope data were derived from the digital elevation model (DEM). Since the topography of the study area did not change significantly in both periods, the same set of topographic data was used for both periods.

The climatic, land use, and topographic factors were, respectively, collected into environmental factor datasets for assessing the habitat suitability of Chinese red pandas at different time periods. Pearson correlation coefficients were used to screen the environmental variables at different time periods, and the variables with a higher ecological relevance were selected for subsequent modeling when two variables were highly correlated (≥0.80). This method can reduce the multicollinearity between environmental variables and ensure that the selected variable set is applicable to both periods. Finally, eight environmental factors were retained for model building. All spatial analyses were performed using ArcGIS 10.5 (ESRI, 2016), and environmental factor correlation analysis was performed in R (R version 4.3.1).

### 2.3. Dynamic Assessment of Habitat Suitability at Multiple Spatiotemporal Scales

To evaluate the change in Chinese red panda habitat suitability, we extracted the Chinese red panda population distribution data from the third and fourth national giant panda survey datasets, respectively. To avoid model overfitting caused by spatial sampling bias, we deleted duplicate records within a 1 km range and finally retained 214 and 309 records of Chinese red panda presence in the two periods, respectively. Analyzing the habitat suitability of the Chinese red panda was conducted by using the distribution data of Chinese red pandas at different time periods and the datasets of environmental variables with the help of the MaxEnt model (version 3.3.3k). We randomly selected 75% of the Chinese red panda distribution data in each period as the training set. The remaining 25% of the data were used as the validation set, and 10-fold cross-validation was used. The average value of the 10 model output results was taken as the giant panda habitat suitability index (HSI). This index has a probability value between 0 and 1; the closer it is to 1 the more suitable the location is for the existence of the species, while the closer it is to 0 the less suitable it is.

The area under the receiving operator curve (AUC) was used to test the model fitting goodness. The higher the AUC value is the better the model prediction ability is. If AUC > 0.8, then it is considered that the model prediction result has accuracy. A map of Chinese red panda habitat quality distribution was created at different time periods based on the model prediction results. The habitat suitability change value (ΔHSI) was calculated by subtracting the predicted HSI value in the fourth survey period from that in the third survey period. ΔHSI was marked as higher than or less than 0 to indicate whether the habitat suitability had increased or decreased.

### 2.4. Analysis of Habitat Suitability Decline in the Liangshan Mountains

A random forest (RF) model was used to analyze the causes of the decline in Chinese red panda habitat suitability in the Liangshan mountains. We generated 1000 random points within the Liangshan mountains and extracted the environmental factors and HSI values of the random points in both periods. The data extracted in the fourth survey period was subtracted from the same point data in the third survey period, and the data with ΔHSI < 0 were retained as the dataset for analyzing habitat suitability decline in Liangshan. ΔHSI was used as the response variable, and the environmental factor change values were used as explanatory variables. Since it was assumed that topographic factors did not change in both periods, they were not included in the RF model analysis. A total of 75% of the dataset was randomly selected as the training set and the remaining 25% as the validation set. The randomForest package was used to build a random forest model. The importance of environmental factors was measured by %IncMSE, where a higher %IncMSE indicated that the variable was more important for model building. The rfpermute package was used to mark the significance level of each environmental factor. For environmental factors with *p* < 0.05, their correlation relationship with the habitat suitability change value was plotted to analyze how their changes affected habitat suitability decline. The random forest model was built in R.

## 3. Results

### 3.1. Habitat Distribution and Habitat Suitability Dynamic Change at Different Time Scales

The average AUC values of the habitat suitability models in the third and fourth survey periods were 0.943 and 0.912, respectively. The model AUC values were both higher than 0.9 and could be used to evaluate the habitat suitability of Chinese red pandas at different time periods.

**Figure 2:**
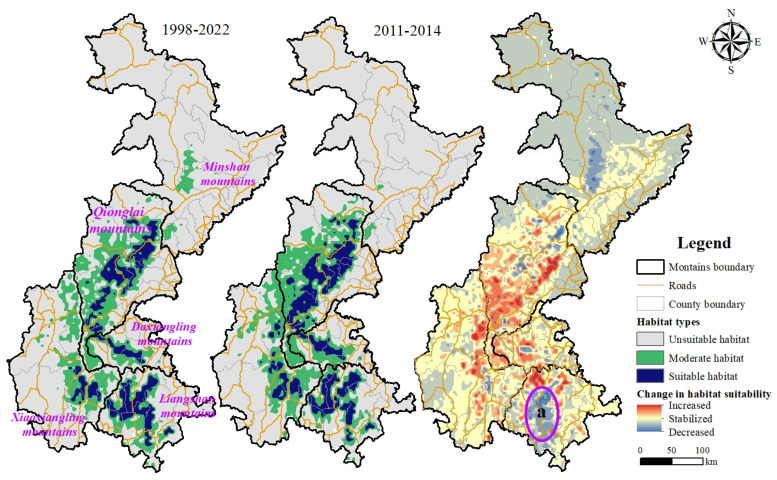
Distribution and change in pattern of the Chinese red panda’s habitat at different times.

The prediction model results show that during the two surveys, the suitable habitat of the Chinese red panda was only distributed in the Qionglai Mountains, Daxiangling Mountains, Xiaoxiangling Mountains, and Liangshan Mountains, among which the Qionglai Mountains and Liangshan Mountains were the mountains where the suitable habitat of the Chinese red panda was concentrated. Generally speaking, the suitable habitat of the Chinese red panda in Sichuan Province presents a belt-like distribution with a high degree of fragmentation. Although the degree of fragmentation of the suitable habitat of the Chinese red panda has decreased during the 10 years of the two surveys, only the habitat patches of the Qionglai Mountains and Daxiangling Mountains show a recovery trend from the perspective of the mountains, while the degree of fragmentation of the habitat patches of the Liangshan Mountains and Xiaoxiangling Mountains has increased (Figure 2, a).

As for the suitability and area of the suitable habitat, the suitability of the Chinese red panda’s habitat in the study area showed varying degrees of increase during the two surveys. The total suitable habitat area increased by 2452.89 km^2^, representing a 27.84% increase compared with the Chinese red panda’s suitable habitat area during the third survey period. The suitable habitat area of the Qionglai Mountains increased by 1622.73 km^2^, showing a 39.63% increase compared with the third survey period. Daxiangling’s and Xiaoxiangling’s suitable habitat areas increased by 230.30 km^2^ and 549.47 km^2^, respectively, indicating increases of 27.78% and 55.53% compared with the third survey period. As one of the primary distribution areas for the Chinese red panda, the suitable habitat area in the Liangshan Mountains increased by 50.39 km^2^, marking a 1.74% increase compared with the third survey period. (Table 2). However, the suitability of Chinese red panda habitats in the Minshan mountains has decreased, and their suitable habitats are disappearing. Although the Liangshan Mountains have shown an increase in suitable habitat areas, the suitability of habitats in some parts of this region has significantly decreased (Figure 2, a).

### 3.2. Analysis of Habitat Suitability Change in the Liangshan Mountains

**Figure 3:**
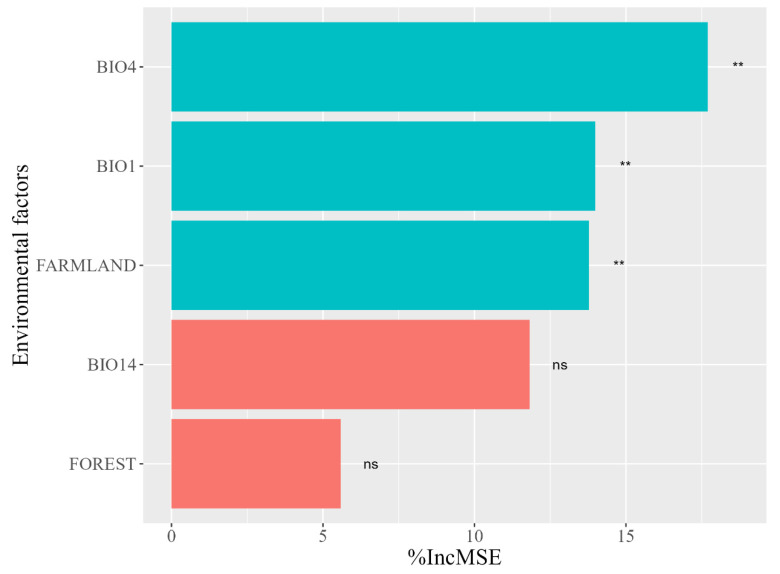
Random Forest model environmental factor importance. BIO1: annual mean temperature; BIO4: temperature seasonality; BIO14: precipitation of driest month; FARMLAND: proportion of cropland area; FOREST: proportion of forest area. “**”: Significance, *p* < 0.01; “ns”: No significance.

The random forest model has a final interpretation of 63.64% for ΔHSI, which can be used to analyze the reasons for the decline in habitat suitability in the central region of the Liangshan Mountains. The model results show that BIO4, BIO1, and FARMALND have a significant impact on the suitability of the habitats in the Liangshan Mountains (*p* < 0.05), with relative importance of 17.64%, 14.22%, and 13.88%, respectively. However, BIO14 and FOREST have no significant impact on the suitability of Chinese red panda habitats in the Liangshan Mountains, with relative importance of 11.86 and 5.63%, respectively (Figure 3).

**Figure 4:**
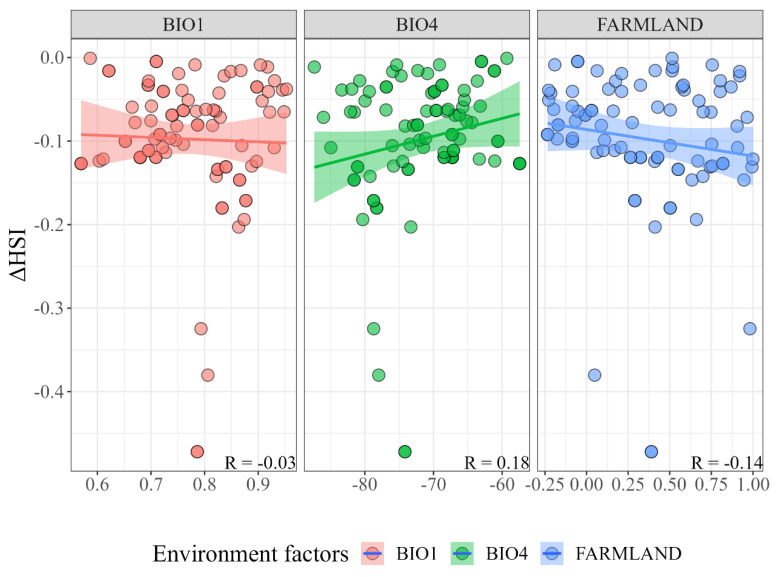
Relationship between environmental factor changes and habitat suitability changes. BIO1: annual mean temperature; BIO4: temperature seasonality; FARMLAND: proportion of cropland area.

The correlation test results of the important environmental factors and the habitat suitability change value showed that the change value of BIO4 was positively correlated with the decline in habitat suitability (R = 0.18), and the change values of BIO1 (R = −0.03) and FARMLAND (R = −0.14) were negatively correlated with the decline in suitability (Figure 4). This indicates that the increase in seasonal temperature difference, environmental temperature, and human activities drove the decline in Chinese red panda habitat suitability in the Liangshan mountains.

## 4. Discussion

Through the analysis of the field survey data of Chinese red pandas, it was found that the suitable habitat for Chinese red pandas was mainly distributed in the Qionglai mountains and Liangshan mountains. The habitat suitability of Chinese red pandas in the study area generally showed different degrees of increases, and the suitable habitat area expanded. However, the habitat suitability of Chinese red pandas in the Minshan mountains declined, and the suitable habitat tended to disappear. Although the Liangshan mountains showed an increase in habitat area, the habitat suitability in the central region declined significantly due to human disturbance and climate change.

### 4.1. Habitat Change at Different Time Scales

A large number of studies have shown that the expansion in the range of human activity is constantly compressing the living environment of wild animals [32,33,34]. The global climate change caused by human activities is also exacerbating the loss of suitable habitats for wild animals [35,36]. Under the current dual pressure of human activity and climate change, the habitat suitability of wild animals generally declines [37,38,39]. Habitat quality, especially for endangered wild animals with high environmental sensitivity, is more likely to decline due to the negative impact of environmental change [40]. Our results show that the habitat suitability of Chinese red pandas shows a general trend of increasing, and the suitable habitat area shows a trend of expansion. The reason may be related to the ecological restoration policies implemented by China in recent years. Since China promulgated the “Law of the People’s Republic of China on the Protection of Wildlife” in 1988, it has successively launched ecological protection projects such as “Returning Farmland to Forest Project” and “Natural Forest Protection Project”. At the same time, Sichuan Province has also established a number of nature reserves with giant pandas as the core protected animals, thus protecting giant pandas and their sympatric species from human activities. Giant pandas and their habitats have been effectively protected, and their population numbers have continued to grow [41]. Chinese red pandas are sympatric with giant pandas, and their habitats may be positively affected by the umbrella effect. Wang et al. (2021) confirmed that China’s protection work on giant pandas and their habitats in the past few decades indirectly achieved the protection of sympatric species [42,43]. Therefore, we believe that the implementation of a series of protection policies has played a positive role in the protection of Chinese red panda populations. Specifically, interference control, the prohibition of logging, vegetation restoration, and other methods reduced human activity’s interference and increased forest coverage [44], thereby improving habitat suitability. Higher canopy cover can provide an ideal microclimate environment for understory species, such as an ideal temperature and relative humidity [20], thus offsetting the impact of widespread climate change. In addition, canopy restoration conducive to the better growth of bamboo forests and understory vegetation [4], thereby providing a stable food source as well as shelter and protection [45,46].

Our analysis shows that macro policies promote the overall improvement of Chinese red panda habitat suitability. However, the habitat suitability of the Minshan mountains declined, and the suitable habitat tended to disappear. Habit disappearance in the Minshan mountains was accompanied by the southward shrinkage of Chinese red panda population distribution, and its northernmost distribution line moved from the northern part of the Minshan mountains to the junction area between the southern part of these mountains and the Qionglai mountains (Figure 1). Generally speaking, population distribution shrinkage is a spatial change that occurs over time under the combined influence of stress factors such as climate, human activities, and the physiological and ecological characteristics of the species [47]. In the current reality scenario, if habitat suitability is generally improved then the question of whether Chinese red pandas still face the risk of persistent population distribution shrinkage becomes one that urgently needs to be answered.

Species often change their distribution to cope with the negative effects of environmental change [48,49]. In recent decades, Chinese red pandas have not shown obvious cluster migrations [5], so there is no evidence to prove that Chinese red pandas have spontaneous population distribution changes. Climate factors are considered to be the key factors driving global species distribution changes [50,51]. Studies have shown that global warming has caused some populations to migrate to higher latitudes to adapt to the gradually rising environmental temperature [52]. However, the direction of Chinese red panda population distribution shrinkage is that toward the low-latitude south. We believe that climate change is not the key driving factor for Chinese red panda population shrinkage because, for Chinese red pandas in the high mountain valleys of the Hengduan Mountains, the environmental regulation function of the forest where their habitat is located can resist the threat of external climate warming [53]. At the same time, adjusting the distribution altitude has also been proven to be one of the ways for forest animals to adapt to changing environments. This is consistent with the conclusion of the study on the distribution area shrinkage of western black-crested gibbons (*Nomascus concolor*) [54]. In addition to climate change, habitat destruction caused by human disturbance is considered to be an important factor affecting species distribution [55]. Habitat degradation and fragmentation lead to population fragmentation, thus forming isolated small populations [56,57]. Inbreeding depression in isolated populations will reduce fertility, juvenile survival rate, and resistance to disease. If they cannot recover or change their living environment through migration, they may be limited by the minimum habitat patch area and minimum population size and become locally extinct [56]. Due to the impact of past forest logging, hunting, and roads, etc., the wildlife habitat in Sichuan Province is patchy. Taking giant pandas as an example, habitat fragmentation has caused their wild populations to be cut off from each other, thus forming 33 local populations of which 24 populations are at risk of extinction [58]. Giant pandas and Chinese red pandas are both food-specialized carnivores, and their habitat environment and food resource selection have similarities [10,59,60]. Due to their close ecological niche, they also suffer from similar environmental stress. It can be inferred that Chinese red pandas in the Minshan mountains may have also experienced habitat destruction and population fragmentation, forming isolated small populations that are isolated from each other and are at a high risk of extinction [61] During the third survey, the encounter rate of Chinese red panda traces in the Minshan mountains was extremely low and the spatial span of the trace points was large (Figure 1), which support our inference. In addition, a high level of inbreeding within isolated populations and external stress (such as infectious diseases) are considered to be the main causes of local population extinction [62]. Therefore, we believe that past human disturbances, such as forest logging, hunting, infrastructure construction, etc., directly caused Chinese red panda habitat fragmentation in the Minshan mountains, thus causing Chinese red panda populations to be separated and isolated. The isolated, small Chinese red panda population in the Minshan mountains may have become locally extinct due to rapid population decline. This also directly led to the distribution shrinkage of Chinese red pandas at the landscape scale.

### 4.2. The Causes of Habitat Suitability Decline

From a wide scale, the habitat suitability of Chinese red pandas shows a general trend of increase, but at a local scale, the habitat suitability of Chinese red pandas in the Minshan and Liangshan mountains shows regional decline. For this reason, we explored the causes of habitat suitability decline. However, this is not enough to support the relevant analysis due to the small number of Chinese red panda trace points in the Minshan mountains, so we only explored the causes of habitat suitability change in the Liangshan mountains. Climate factors are usually considered to be the key indicators for assessing habitat suitability and are also important factors for species occurrence [63]. Due to the high vulnerability of endangered wildlife to climate change, they are more susceptible to climate change [64,65]. In recent decades, the climate of giant panda and Chinese red panda habitats has become warmer and drier, and this pattern of climate warming will continue [66]. This is also the most critical driving factor for the dynamic change of population distribution of sympatric species of giant pandas [67,68]. We found that climate change caused the habitat suitability change of Chinese red pandas in the Liangshan mountains, which mainly manifested itself through the increase in environmental temperature and the enhancement of temperature seasonality in the habitat, which are consistent with the current direction of climate change. In addition, related studies on Chinese red panda habitat selection in the Himalayan region also showed that Chinese red pandas responded strongly to temperature- and precipitation-related bioclimatic variables [69]. These studies support the impact of climate change on Chinese red panda habitat suitability. However, on a local scale, the habitat suitability change in the Liangshan mountains is opposite to the trend at the landscape scale. Compared with the Qionglai, Daxiangling and Xiaoxiangling mountains, the Liangshan mountains still suffered from the negative impact of climate change under the implementation of ecological protection policies, thus indicating that the Chinese red panda’s habitat in the Liangshan mountains has ecological fragility [47,48]. This may be related to the poor vegetation restoration status and long-term existence of grazing and other human disturbances in the Liangshan mountains for many years. The Liangshan mountains experienced a long period of forest logging and was one of the most severely damaged areas [26]. In the 1980s, timber was an important economic resource in the Liangshan mountains [70], and forest logging caused a sharp decline in vegetation coverage in the Liangshan mountains. Although it has been restored by natural or artificial intervention for many years, due to the regional dry climate and the single tree species for restoration planting, most areas have a slow forest structure and function restoration process and find it difficult to resist the negative impact of climate change. Although various ecological protection projects were implemented after 2000 and forest logging was prohibited, animal husbandry gradually replaced timber sales as the main source of income for some residents in the Liangshan mountains, which is the main disturbance to wildlife habitats in the Liangshan mountains [71]. In addition, collection activities are also common in the mountains [3]. Although there is no direct competition for food resources between livestock and Chinese red pandas [14], grazing behavior will cause Chinese red pandas’ avoidance behavior [72,73], and grazing activities will also aggravate habitat degradation through trampling [74,75]. In summary, grazing activities may be the main reason for the decline in Chinese red panda habitat quality in the central region of the Liangshan mountains. Due to the low vegetation coverage in some areas of Liangshan mountains, climate change may also have a negative impact on the Chinese red panda’s habitat.

## 5. Conclusions

This study underscores the critical importance of dynamic species habitat research in formulating effective conservation and management strategies. Chinese red pandas predominantly inhabit the Qionglai and Liangshan mountains, with an overall increasing trend in habitat suitability and expanded suitable areas. However, a concerning decline in habitat suitability and the potential disappearance of the suitable habitat were observed in the Minshan mountains. While global climate change and human activities, such as ecological restoration policies, have contributed to the overall improvement in Chinese red panda habitat suitability, local-scale challenges persist. Particularly in the central region of the Liangshan mountains, negative impacts from human disturbances, notably those from grazing activities, have led to a decline in habitat quality. Furthermore, the local extinction of isolated Chinese red panda populations in the Minshan mountains has resulted in distribution retreat.

## 6. Protection Management Proposal

Based on the research findings, we believe that the future conservation and management of Chinese red panda habitats should be tailored according to the current conditions of these habitats across different mountainous regions. For instance, in the Liangshan Mountains, measures such as controlling grazing and other human disturbances should be implemented. Additionally, ecological restoration through artificial interventions should be undertaken to restore the structure and functionality of ecosystems and enhance habitat quality. Given the decline in Chinese red panda populations in the Minshan mountains region due to local extinctions, we recommend reintroducing Chinese red pandas to facilitate population recovery.

## Figures and Tables

**Table 1:** Environmental factors for assessment of Chinese red panda habitat suitability.

Factor Type	Environmental Factors	Description	Unit
Climate	BIO1	Annual Mean Temperature	°C
	BIO4	Temperature Seasonality	-
	BIO14	Precipitation of Driest Month	mm
Terrain	ELEVATION	Mean Elevation	m
	SLOPE	Mean Slope	°
Land Use	FARMLAND	Proportion of Cropland Area	%
	FOREST	Proportion of Forest Area	%
	ROAD	Distance to Main Road	m

**Table 2:** Changes in suitable habitat area for Chinese red pandas at different times.

Mountains	3rd Survey Area (km^2^)	4th Survey Area (km^2^)	ΔArea (km^2^)	Relative Change (%)
Minshan	0.00	0.00	0.00	0.00
Qionglai	4094.97	5717.70	1622.73	39.63
Daxiangling	828.92	1059.22	230.30	27.78
Xiangxiangling	989.47	1538.94	549.47	55.53
Liangshan	2898.68	2949.07	50.39	1.74
Total	8812.04	11,264.93	2452.89	27.84

## Data Availability

Data are contained within the article.
